# Cellular and subcellular localization of Marlin-1 in the brain

**DOI:** 10.1186/1471-2202-10-37

**Published:** 2009-04-22

**Authors:** René L Vidal, José I Valenzuela, Rafael Luján, Andrés Couve

**Affiliations:** 1 Fisiología y Biofísica, Instituto de Ciencias Biomédicas, Facultad de Medicina, Universidad de Chile, Independencia 1027, Santiago, Chile; 2 Nucleus of Neural Morphogenesis (NEMO), Faculty of Medicine, Universidad de Chile, Santiago, Chile; 3 Universidad Austral de Chile, Independencia 567, Isla Teja, Valdivia, Chile; 4 Centro Regional de Investigaciones Biomédicas, Departamento de Ciencias Médicas, Facultad de Medicina, Universidad Castilla-La Mancha, 02006 Albacete, Spain

## Abstract

**Background:**

Marlin-1 is a microtubule binding protein that associates specifically with the GABA_B1 _subunit in neurons and with members of the Janus kinase family in lymphoid cells. In addition, it binds the molecular motor kinesin-I and nucleic acids, preferentially single stranded RNA. Marlin-1 is expressed mainly in the central nervous system but little is known regarding its cellular and subcellular distribution in the brain.

**Results:**

Here we have studied the localization of Marlin-1 in the rodent brain and cultured neurons combining immunohistochemistry, immunofluorescence and pre-embedding electron microscopy. We demonstrate that Marlin-1 is enriched in restricted areas of the brain including olfactory bulb, cerebral cortex, hippocampus and cerebellum. Marlin-1 is abundant in dendrites and axons of GABAergic and non-GABAergic hippocampal neurons. At the ultrastructural level, Marlin-1 is present in the cytoplasm and the nucleus of CA1 neurons in the hippocampus. In the cytoplasm it associates to microtubules in the dendritic shaft and occasionally with the Golgi apparatus, the endoplasmic reticulum (ER) and dendritic spines. In the nucleus, clusters of Marlin-1 associate to euchromatin.

**Conclusion:**

Our results demonstrate that Marlin-1 is expressed in discrete areas of the brain. They also confirm the microtubule association at the ultrastructural level in neurons. Together with the abundance of the protein in dendrites and axons they are consistent with the emerging role of Marlin-1 as an intracellular protein linking the cytoskeleton and transport. Our study constitutes the first detailed description of the cellular and subcellular distribution of Marlin-1 in the brain. As such, it will set the basis for future studies on the functional implications of Marlin-1 in protein trafficking.

## Background

Marlin-1 (Jamip-1, Jakmip1) is a 73 kDa protein that interacts directly with metabotropic GABA_B _receptors in neurons [1] and with members of the Janus kinases family (Jaks) in lymphoid cells [2]. Marlin-1 is highly conserved in vertebrates and its structure consists of three coiled-coil domains containing two leucine zippers [1]. A 200 aminoacid region in the C-terminus is responsible for binding GABA_B1 _and Jaks [1,2]. A central region binds RNA *in vitro *[1] and the N-terminus interacts with microtubules and kinesin-I [2,3]. The N-terminus is also involved in the stabilization of the microtubular network in cell lines, specifically opposing the destabilizing effect of Nocodazole [2]. Marlin-1 co-sediments with a cytoskeleton complex in the brain and moves in a microtubule-dependent manner in dendrites of hippocampal neurons [3].

Five splice variants for Marlin-1 (a-e) have been described recently [4]. Expression of Marlin-1a has been reported preferentially in the central nervous system (CNS), but additional expression has been observed in lymphoid cells, testis and skeletal muscle [2,4,5]. Marlin-1b, 1c and 1d are predominantly expressed in the brain, whereas Marlin-1e is not present in the brain but is abundant in lung and liver.

Marlin-1 has been associated with carotid body morphology and function, and Crohn's disease [6,7]. Furthermore, altered expression of Marlin-1 has been reported in two genetic conditions linked to autism [8]. However, despite its potential role in this severe neurological pathology the specific cellular and subcellular localization in the CNS has not been reported [1,2]. In addition, it has been suggested that association to the microtubule cytoskeleton constitutes a central aspect of Marlin-1 function, but a high resolution microscopic analysis of the association of Marlin-1 and microtubules in the brain is missing [2,3].

Here we describe the cellular and subcellular localization of Marlin-1 in the brain. We study the cellular localization of Marlin-1 in the rodent brain via immunohistochemistry and the subcellular localization of Marlin-1 in the adult rat hippocampus using pre-embedding electron microscopy (EM). We complement these studies with immunofluorescence in hippocampal neurons. Our results demonstrate that Marlin-1 is abundant in restricted regions of the brain including olfactory bulb, cerebral cortex, hippocampus, medulla, pons and cerebellum. Marlin-1 is a neuronal specific protein present in dendrites and axons, but only occasionally in dendritic spines. Marlin-1 is associated to microtubules in neurites and to a lesser extent to intracellular organelles in the cell body. It is also associated to euchromatin in the nucleus. These observations are consistent with the proposed function of Marlin-1 relative to transport and the cytoskeleton.

## Results

### Marlin-1 is expressed in restricted regions of the CNS

**Figure 1:**
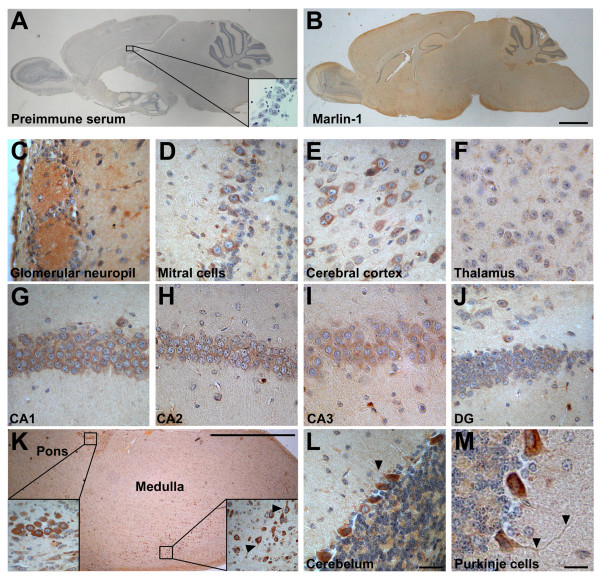
**Cellular distribution of Marlin-1 in the brain**. (A) Sagittal section of a mouse brain showing immunoperoxidase staining with pre-immune serum. Inset shows a high magnification image of the hippocampus. (B) Same as above with Marlin-1 antibodies. (C-M) High magnification images of regions of interest: (C) glomerular neuropil of olfactory bulb; (D) mitral cells of olfactory bulb; (E) layer IV of cerebral cortex; (F) thalamus; (G) CA1 of hippocampus; (H) CA2 of hippocampus; (I) CA3 of hippocampus; (J) dentate gyrus; (K) brainstem; insets show higher magnification of indicated areas; (L) Purkinje cells of the cerebellum; (M) higher magnification of Purkinje neurons of the cerebellum. Scale bar in B, 100 μm (same for A and B); scale bar in L, 20 μm (same for C-J and L); scale bar in K, 100 μm; scale bar in M, 10 μm.

To determine the cellular distribution of Marlin-1 in the CNS brain slices were prepared from adult mouse and subject to immunohistochemistry using pre-immune or Marlin-1 antibodies [1]. No specific staining was detected with pre-immune antibodies (Fig. 1A and inset). On the contrary, a specific labeling was observed with Marlin-1 antibodies (Fig. 1B). Although at low magnification the distribution of the immunostaining appeared relatively uniform, higher magnification images demonstrated that the protein localized to specific cellular groups in discrete areas of the brain (Figs. 1C–M and Table 1). Prevalent regions included the olfactory bulb, cerebral cortex, hippocampus, hipothalamus, preoptic area, medulla and cerebellum. Little expression of Marlin-1 was observed in the corpus callosum and anterior commissure (not shown).

**Table 1:** Semiquantitative analysis of the cellular distribution of Marlin-1 in the brain

**Brain Area**	**Relative Abundance**
**Cerebral Cortex**	
Layers I-II	++
Layers III-IV	+++
Layers IV-VII	++

**Corpus Callosum**	-

**Olfactory Bulb**	
Glomerular Layer	+++
Mitral Cell Layer	+++
Internal Granule Cells	++

**Hippocampus**	
CA1 Region	+++
CA2 Region	+++
CA3 Region	+++
Dentate Gyrus	++

**Thalamus**	++

**Hypothalamus**	
Dorsomedial Hypothalamic Nucleus	+++
Ventromedial Hypothalamic Nucleus	+++

**Preoptic Area**	+++

**Fornix**	-

**Nucleus Accumbens**	++

**Anterior Commissure**	-

**Midbrain**	
Superior Colliculus	+
Inferior Colliculus	+
Lateral Periaqueductal Grey	+

**Pons**	
Pontine Nuclei	++
Pontine Reticular Nucleus	+

**Medulla**	
Medial Vestibular Nucleus	+++
Medullary Reticular Nucleus	+
Gigantocellular Reticular Nucleus	+++

**Cerebellum**	
Molecular Layer	+
Purkinje Cells	+++
Granular Layer	++

Abundance of Marlin-1 in different brain regions was determined from immunohistochemistry of the sagital sections of the mouse brain. Brain areas were characterized according to its level of abundance of Marlin-1 as: high level (+++), medium level (++), low level (+) and very low level (-).

In the olfactory bulb the staining was prominent in the glomerular neuropil and the mitral cell body layer (Figs. 1C–D). Marlin-1 was not observed in neurons of the external or internal plexiform layer of the olfactory bulb. In the cerebral cortex Marlin-1 was present in all cortical layers, but mostly in layers III and IV (Fig. 1E). The staining was less prominent in other brain areas such as the thalamus (Fig. 1F). In the hippocampus, cells showed high expression of Marlin-1 in CA1, CA2 and CA3 pyramidal cell layers, being less prominent in the granular cell layer of the dentate gyrus (Figs. 1G–J). Expression was moderate in the pons region and high in several nuclei of the medulla including in the area of the medial vestibular nucleus and the gigantocellular reticular nucleus (Fig. 1K and inset). In the cerebellum Marlin-1 was highly expressed in Purkinje cells (Figs. 1L and 1M). The glomerular and molecular layers of the cerebellum showed lower levels of Marlin-1. The staining for Marlin-1 was intense in the soma in all the areas analyzed, but neurites were also labeled. This was particularly evident in the medulla and Purkinje cells of the cerebellum (Figs. 1K, L and 1M arrowheads).

### Marlin-1 is a neuronal specific protein

Although initially described in hippocampal neurons, many aspects of the cellular and subcellular localization of Marlin-1 using light and electron microscopy remain unknown [1]. Importantly, the suitability of the hippocampus and its cultured neurons to evaluate the localization of Marlin-1 is supported by the abundant expression of the transcript and protein for Marlin-1 in this brain area [9].

**Figure 2:**
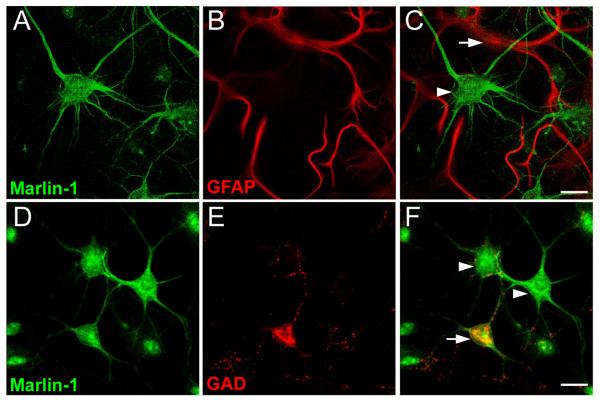
**Cellular distribution of Marlin-1 in hippocampal cultures**. (A-C) Neurons were fixed at 14 div and co-labeled with antibodies to Marlin-1 (green) and GFAP (red). (D-F) Same as above for Marlin-1 and GAD. Merge images are shown on the right. Scale bars, 10 μm (A-F).

First, to examine the distribution of Marlin-1 in specific cellular types we carried out a light microscopy analysis in primary hippocampal cultures. Marlin-1 was only observed in neuronal cell types and was absent from glia as indicated co-labeling with GFAP, a specific glial marker (Figs. 2A–C, GFAP-positive, arrow; Marlin-1-positive/GFAP-negative, arrowhead). To determine the identity of neurons that expressed Marlin-1 we used GAD, an established marker for GABAergic interneurons. Marlin-1 was present in GAD-positive and GAD-negative neurons (Figs. 2D–F, GAD-positive, arrow; GAD-negative, arrowheads). Combined, these results indicate that Marlin-1 is a neuronal specific protein expressed in interneurons and pyramidal neurons in the hippocampus.

**Figure 3:**
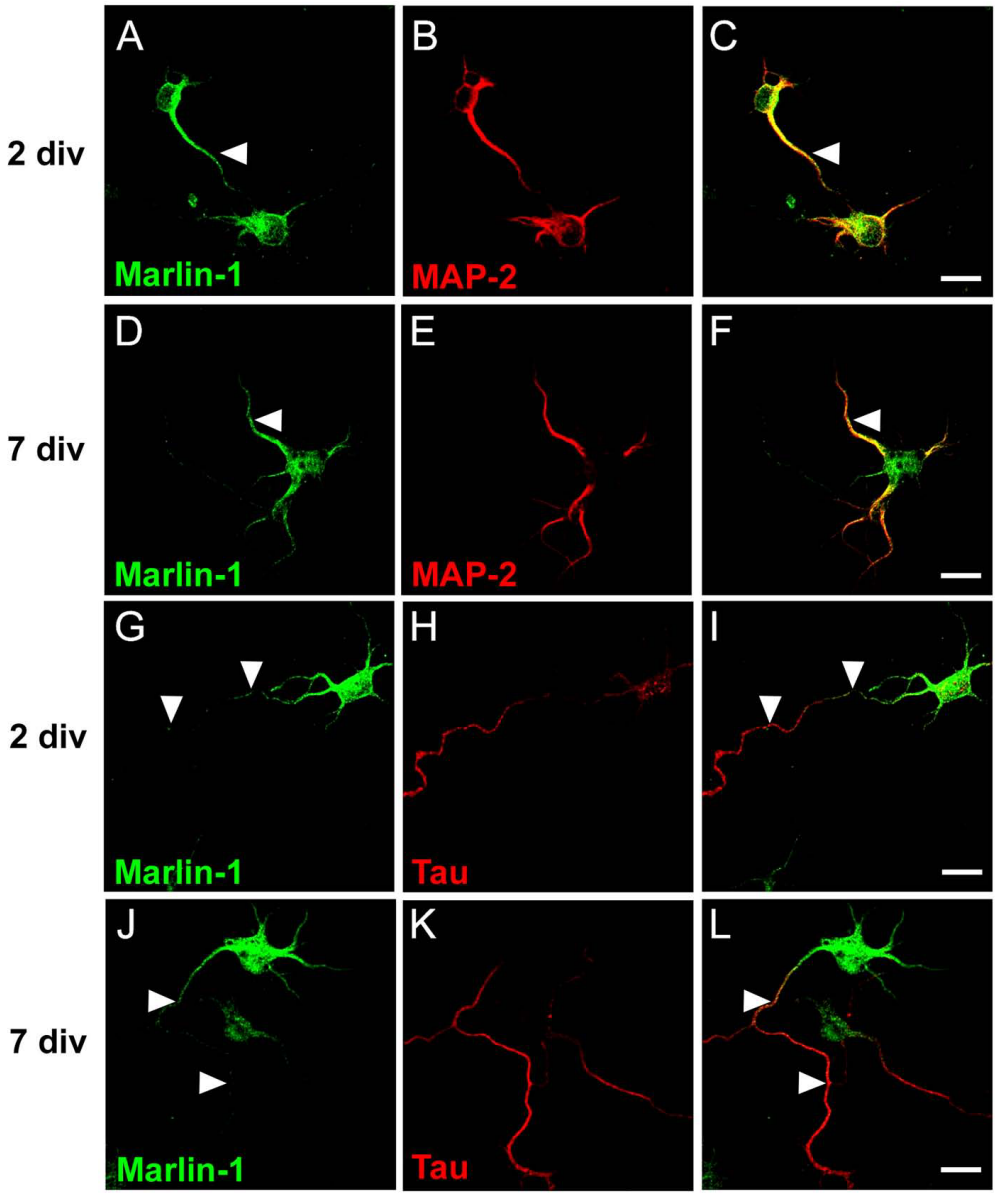
**Subcellular distribution of Marlin-1 in neurites of hippocampal neurons**. (A-C) Neurons were fixed at 2 div and co-labeled with antibodies to Marlin-1 (green) and MAP2 (red). (D-F) Same as above for neurons fixed at 7 div. (G-I) Neurons were fixed at 2 div and co-labeled with antibodies to Marlin-1 (green) and Tau (red). (J-L) Same as above for neurons fixed at 7 *div*. Merge images are shown on the right. Scale bars, 20 μm (A-L).

We then determined the type of neurites that contained Marlin-1 by using MAP2 and Tau, established dendritic and axonal markers respectively. Marlin-1 was present in dendrites and axons of stage 3 and 5 hippocampal neurons [10]. In dendrites, Marlin-1 was prominent in the proximal region and gradually diminished towards distal neurites resembling MAP2 (Figs. 3A–F). In axons, the majority of neurons displayed a strong immunoreactivity in the axon initial segment that gradually decreased distally (Figs. 3G–L, arrowheads). Occasionally, a weaker staining for Marlin-1 was observed in distal portions of the axon (not shown). Similar results were obtained at different developmental stages (2–21 div, not shown). These results conclusively demonstrate that Marlin-1 is a neuronal specific protein with dendritic and limited axonal distribution.

### Subcellular localization of Marlin-1

**Figure 4:**
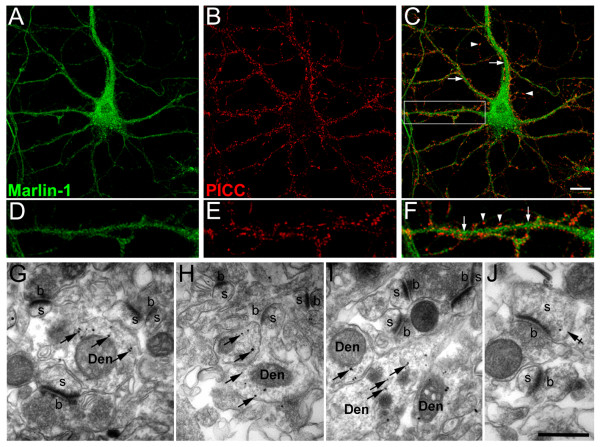
**Localization of Marlin-1 relative to synapses**. (A-C) Cultured hippocampal neurons were fixed at 14 div and co-labeled with antibodies to Marlin-1 (green) and Piccolo (PICC, red). (D-F) High magnification of the area boxed in C. Scale bars, 10 μm (A-C). Arrows, neurite axis; arrowheads, synapses. (G-J) Pre-embedding immunogold EM of the CA1 region of the hippocampus incubated with Marlin-1 antibodies. Marlin-1 is found in the cytoplasm of dendritic shafts (Den, arrows), and occasionally in dendritic spines (s, crossed arrow) but not in presynaptic boutons (b). Scale bar, 0.5 μm (G-J).

To begin exploring the subcellular distribution we assessed the synaptic localization of Marlin-1 using Piccolo, a protein that concentrates in presynaptic boutons [11]. Marlin-1 localized within the cell body and along the axis of neurites. Some apposition between Piccolo-positive structures and Marlin-1 was visible in dendrites, but the majority of the protein did not colocalize with Piccolo. In contrast, it distributed to a more central area of the dendritic shaft (Figs. 4A–C and higher magnification D-F. Neurite axis, arrows; synapses, arrowheads). To confirm these findings the synaptic distribution of Marlin-1 was examined at the EM level using pre-embedding immunogold labeling in the CA1 region of hippocampus. In agreement with our light microscopy analysis Marlin-1 was frequently present in the cytoplasm of dendrites (Figs. 4G–J, arrows), but only occasionally associated to dendritic spines (Figs. 4J, crossed arrow). No immunoreactivity for Marlin-1 was detected in synaptic boutons (Figs. 4G–J, b).

**Figure 5:**
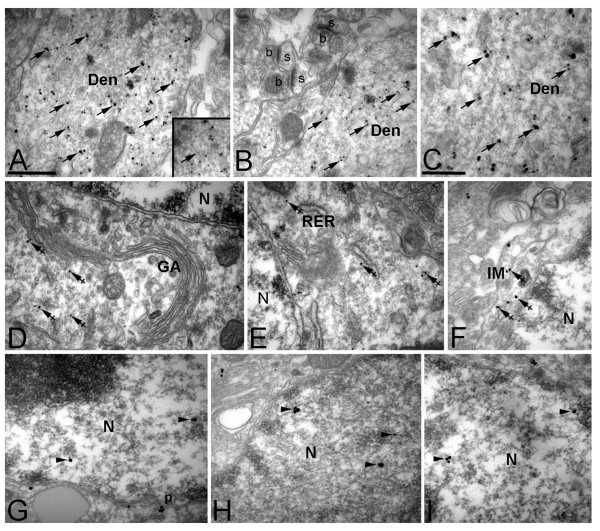
**Subcellular localization of Marlin-1 in the CA1 region of the hippocampus relative to microtubules, secretory organelles and the nucleus**. (A-C) Pre-embedding immunogold EM of the CA1 region of the hippocampus incubated with Marlin-1 antibodies. Immunoparticles are localized at intracellular sites mainly associated to microtubules in the dendritic shaft (arrows). (D-F) Immunoparticles are frequently found near cisternae of the Golgi apparatus (GA), the rough endoplasmatic reticulum (RER) (crossed arrows) and other intracellular membranes (IM). (G-I) Marlin-1 is present in the nucleus (N) associated with euchromatin (arrowheads). Localization of Marlin-1 is also evident near the perikaryon (p). Scale bar in C, 0.5 μm (same for A, B, D-I) and scale bar in D, 0.2 μm.

We also used pre-embedding immunogold EM to examine the subcellular distribution of Marlin-1 away from synapses. Marlin-1 was present in many thick and thin dendritic profiles that were often traced back to their origin in pyramidal and nonpyramidal cells. In spine bearing dendrites of pyramidal cells, cytoplasmic labeling was present in thick trunks (>0.5 μm diameter) of apical and basal dendrites, and in thin branches (<0.5 μm diameter) (Figs. 5A–C, and Figs. 4G–J). More importantly, in dendritic shafts Marlin-1 was frequently associated to electron dense structures corresponding to microtubules (Figs. 5A–C, arrows). We next evaluated the subcellular localization of Marlin-1 relative to secretory organelles in neuronal somata. In the cytoplasm Marlin-1 was not a major component of the stacked cisternae of the Golgi apparatus or the rough ER, but was observed in the vicinity of these secretory organelles and other intracellular membranes (Figs. 5D–F, crossed arrows). The physical proximity between a fraction of Marlin-1 and the ER and Golgi apparatus was also observed by fluorescent microscopy in mature hippocampal neurons [see Additional File 1]. Finally, a proportion of Marlin-1 appeared in the nucleus, preferentially associated with euchromatin and the nuclear membrane. Clusters of immunoparticles for Marlin-1 were frequently observed in these regions (Figs. 5G–I, arrowheads). Importantly, no specific staining was observed in any of the regions mentioned when a pre-immune serum was used (not shown).

## Discussion

### Marlin-1, the cytoskeleton and secretory organelles

Our EM analysis and previous biochemical data [3] provide strong evidence that Marlin-1 is associated to the microtubule cytoskeleton in the brain. The association of Marlin-1 to microtubules has been reported previously in Jurkat cells, neurons and testis [2,3,5]. In addition, the interaction with molecular motors has been demonstrated in brain extracts [3]. Interestingly, we have been unable to obtain a reproducible association of Marlin-1 with microtubules in COS-7 cells, an epithelial fibroblast-like cell line (not shown). This difference has not been explained satisfactorily, but it is possible that Marlin-1 interacts with a specific tubulin isotype not present in COS-7 cells, such as β(III)-tubulin. Thus, the precise mechanism and molecular specificity of Marlin-1 and microtubule binding awaits clarification.

We occasionally observe the accumulation of Marlin-1 in a perinuclear region in hippocampal neurons (not shown) and our EM analysis indicates that a proportion of endogenous Marlin-1 associates to the rough ER and Golgi in the brain. Recently, two proteins belonging to the Marlin-1 family, namely NECC1 and NECC2, have been identified in frogs (*Rana ridibunda*) [12]. These correspond to the mammalian orthologs Jamip2 and Jamip3 [2]. Jamip2 (NECC1) and Jamip3 (NECC2) are abundant in the brain, are structurally similar to Marlin-1 and consist on a series of long coiled-coil domains interspersed with non-coiled-coil stretches. Contrary to Marlin-1 they contain a transmembrane domain at their C-termini [2,13]. Cruz-Garcia et al. (2007) suggest that these proteins may be considered part of the diverse family of Golgin tethering factors. Golgins organize the Golgi apparatus and regulate membrane trafficking through their interaction with the cytoskeleton and cytoskeletal motors [13]. Jamip2-NECC1 and Jamip3-NECC2 colocalize partially with the Golgi apparatus upon over-expression in HEK-293 cells, but their distribution differs from GM130, a marker for CGN and cis Golgi stacks, and colocalization with VSVG-GFP ts045 is weak [12]. Nevertheless, Jamip2-NECC1/Jamip3-NECC2 and the Golgi coincide in a perinuclear region. Regarding Marlin-1 more experiments are needed to determine whether it may also be considered part of the Golgin family, but it will be of interest to further examine its role in protein trafficking in the context of its distribution relative to microtubules and secretory organelles.

A small proportion of Marlin-1 is present in dendritic spines, but not in presynaptic terminals in the hippocampus. However, the strong axonal staining observed in hippocampal neurons using immunofluorescence and the labeling of olfactory bulb glomeruli should be taken into account when characterizing the distribution of Marlin-1 in axon terminals, especially in the olfactory system.

Overall, the cellular pattern of protein expression of Marlin-1 coincides with the in situ hybridization data reported in the Allen Mouse Brain Atlas [9]. However, some discrepancies exist in restricted regions of the brain such as dentate gyrus. These differences probably reflect translational regulation, differential protein stability or preferential expression of specific splice variants.

### A proportion of Marlin-1 is nuclear

Our EM and immunofluorescence analyses show that a proportion of Marlin-1 is nuclear. These results are consistent with nuclear localization and nuclear export signals present in the protein and support previous studies regarding the interaction of Marlin-1 with nucleic acids *in vitro*, in particular with single stranded RNA [1]. It is currently unknown whether the cytoskeletal and nuclear localizations of Marlin-1 reflect two separate roles of Marlin-1 or are two aspects of a single function. Likewise, it remains to be studied whether different splice variants of Marlin-1 account for the different cellular and subcellular distribution patterns observed in the present study [4]. The fact that the antibodies used here detect all but one of the splice variants requires this possibility to be examined promptly.

### Functional implications of the distribution of Marlin-1

Marlin-1 was originally identified as a GABA_B _receptor and Jaks interacting protein [1,2]. Moreover, a general cytoskeletal function has appeared [1-3,5]. Here we have focused exclusively on the distribution of Marlin-1 to provide an unbiased analysis of the protein in the brain, and particularly in the hippocampus.

Our studies are consistent with the emerging role of Marlin-1 as an intracellular protein linking the cytoskeleton and transport in neurons and lymphocytes. In neurons, GABA_B _receptors have been found in association with microtubules and intracellular organelles, and their highly regulated trafficking make them ideal targets for a transport system linking secretory organelles and the cytoskeleton [14-18]. In CD8+ T lymphocytes, the transport of secretory granules to the cell periphery and the regulation of cytotoxic activity [19] is also a system likely to benefit from organelle-cytoskeleton linking proteins that bind different partners through distinct modular domains.

## Conclusion

In the present study we provide the first detailed description of the cellular and subcellular distribution of Marlin-1 in the brain. We have used immunohistochemistry to evaluate the cellular distribution of Marlin-1. We have also employed immunofluorescence and EM to examine its subcellular distribution. Marlin-1 is present exclusively in neuronal cell types. Marlin-1 positive neurons are located in several brain regions, such as olfactory bulb, hippocampus, cerebral cortex, brainstem and cerebellum. The expression of the protein is abundant in GABAergic and non GABAergic cells. It is concentrated in the shaft of dendrites and the axon initial segment but less prominent in dendritic spines. Marlin-1 is associated to the microtubule cytoskeleton, a proportion is related to membranes of secretory organelles and a significant pool is found in the neuronal nucleus. Our results will set the basis for future analyses of the functional implications of Marlin-1 in protein trafficking in neurons.

## Methods

### Antibodies

Antibodies directed against the N- and C-terminal domains of Marlin-1 have been reported before [1]. We have previously characterized their specificity and reported adsorption controls to demonstrate their suitability for localization studies [1,3]. Here we have used N-terminus antibodies directed against aminoacids 1–374 that recognize a doublet in brain and neuronal preparations [1]. These antibodies recognize all the splice variants of Marlin-1 except Marlin-1E [4]. Since Marlin-1E has not been found in the brain, our study takes into account all the relevant neuronal splice variants. Marlin-1 antibodies (0.3 μg/μl) were used at a dilution of 1:300 for immunohistochemistry and immunofluorescence, and 1:50 for pre-embedding immunoelectron microscopy. Glial fibrillary acidic protein (GFAP) antibodies were purchased from Santa Cruz Biotechnology (Santa Cruz, CA) and used at a dilution of 1:500. Piccolo antibodies were kindly provided by E.D. Gundelfinger and W.D. Altrock (Leibniz Institute for Neurobiology, Magdeburg, Germany) and used at a dilution of 1:200. Glutamic acid decarboxylase (GAD), MAP2 and Tau antibodies were purchased from Chemicon (Temecula, CA) and used at a dilution of 1:500. KDEL antibodies (Grp78, BiP) were purchased from StressGen (Ann Arbor, MI) and used at a dilution of 1:200. GM130 antibodies were purchased from BD Transduction Laboratories (Palo Alto, CA) and used at a dilution of 1:500. Secondary anti-mouse, anti-rabbit or anti-guinea pig antibodies were purchased from Jackson Immuno Research Laboratories (West Grove, PA). Secondary antibodies conjugated to Texas Red (TR), tetramethyl rhodamine isothiocyanate (TRITC), fluorescein isothiocyanate (FITC) were used at a dilution of 1:500, and horseradish peroxidase antibodies (HRP) were used at a dilution of 1:5000 respectively. Secondary anti-rabbit antibodies coupled to 1.4-nm gold particles were purchased from Nanoprobes (Stony Brook, NY).

### Animals

Adult pregnant female Sprague-Dawley rats and adult BALB/c mice were purchased from the Central Animal Facility at Universidad Católica de Chile and killed by asphyxia in a CO_2 _chamber according to the Guide for Care and Use of Laboratory Animals (copyright 1996, National Academy of Science).

### Neuronal cultures

Primary hippocampal neurons were cultured from E18 rats as reported [20].

### Immunohistochemistry

To simultaneously preserve delicate tissue and antigenicity, mice were deeply anesthetized, brains were rapidly removed from animals and fixed in 4% (v/v) Bouin's fluid for 24 h at RT. This procedure compared positively to fixation in paraformaldehyde. After dehydration in ethanol series, tissues were embedded in Paraplast Plus (Monoject Scientific, Saint Louis, MO) or Hiscosec (Merck, Darmstadt, Germany). 50 μm sections were processed for deparaffinization with alcohol series and incubated in 10% H_2_O_2 _for 15 min. Immunostaining was performed using the Universal ICQ LSAB plus kit (DAKO, Glostrup, Denmark). Sections were rinsed in H_2_O and phosphate buffer saline (PBS) for 10 min and incubated with primary antibody at 22°C overnight. Sections were washed three times in PBS and incubated with anti-rabbit IgG-biotin conjugated secondary antibody for 25 min at RT. Sections were then washed three times in PBS and incubated with streptavidin/peroxidase anti-peroxidase complex for 25 min at RT. Sections were washed three times in PBS and developed with diaminobenzidine for 5 min. Sections were then rinsed with H_2_O to stop the reaction and counterstained with hematoxylin for 30 sec at RT. Finally, sections were incubated with borate and dehydrated with a series of alcohols before mounting. Coverslips were examined using a Zeiss Axioskope II microscope equipped with a digital video camera (Nikon DXM1200).

### Pre-embedding immunoelectron microscopy

Rats were deeply anesthetized and perfused with 4% paraformaldehyde, 0.2% picric acid, and 0.05% glutaraldehyde in 0.1 M phosphate buffer (PB, pH 7.4). Coronal, 60 μm sections were cut with a Vibratome and collected in 0.1 M PB. Sections were incubated in 10% normal goat serum (NGS) in 50 mM Tris buffer (pH 7.4) containing 0.9% NaCl (TBS), for 1 h. Sections were then incubated for 24 h with polyclonal antibodies against Marlin-1 at a final protein concentration of 1 μg/ml in TBS containing 1% NGS. After washes in TBS, sections were incubated for 2 h in goat anti-rabbit coupled to 1.4-nm gold particles (Nanoprobes, Stony Brook, NY) diluted 1:100 in TBS containing 1% NGS. After several washes in PBS, sections were postfixed in 1% glutaraldehyde dissolved in the same buffer. They were washed in double distilled water, followed by silver enhancement of the gold particles with an HQ Silver kit (Nanoprobes, Stony Brook, NY). The gold-silver-labeled sections were treated with OsO_4 _(1% in 0.1 M PB), block-stained with uranyl acetate, dehydrated in a graded series of ethanol and flat-embedded on glass slides in Durcupan resin (Fluka, Reidel-deHaen, UK). Regions of interest were cut at 70–90-nm-thick sections with an ultramicrotome (Reichert Ultracut E, Leica, Austria). Ultrathin sections were contrasted with lead citrate and analyzed in a Jeol-1010 electron microscope.

### Immunofluorescence and fluorescent microscopy

Glass coverslips with attached neurons were fixed for 10 min in a PBS solution containing 4% paraformaldehyde and 4% sucrose. Cells were permeabilized by incubating 10 min in PBS containing 0.5% bovine serum albumin (BSA) and 0.5% Nonidet P-40, blocked in PBS containing 0.05% BSA, 10% horse serum and stained with primary antibodies overnight at 4°C and with secondary antibodies for 1 h at RT before mounting them with Vectashield (Vector Labs, Burlingame, CA).

Images were acquired using a Zeiss LSM-5, Pascal 5 Axiovert 200 confocal microscope (Plan-Apochromat 63x/1.4 oil DIC objective) and LSM 5 3.2 image capture and analysis software. Alternatively images were obtained using an Olympus BX61WI upright microscope with an Olympus DSU spinning disk unit (UPlan FL N 60x/1.25 oil iris objective). Raw images were deconvolved by Huygens Scripting software (Scientific Volume Imaging, Hilversum, Netherlands) using the Classic Maximum Likelihood Estimator algorithm.

## Authors' contributions

RLV carried out immunohistochemistry, immunofluorescence and drafted the manuscript. JIV contributed to immunofluorescence and helped to draft the manuscript. RL carried out electron microscopy in collaboration with RLV. AC conceived the study, participated in its design and coordination, and helped to complete the draft. All authors read and approved the final manuscript.

## Supplementary Material

Additional file 1 **Subcellular distribution of Marlin-1 in cultured hippocampal neurons relative to secretory organelles**. Neurons were fixed at 8 div and stained with Marlin-1 antibodies (A and D) and with Golgi matrix (B) or ER antibodies (E). FITC or TR conjugated secondary antibodies were used to visualize immunostaining. Merge panels are shown on the right (C and F). Click here for file
