# Supplementary figures and images for: Cellular and subcellular localization of Marlin-1 in the brain
Source: BMC Neurosci. 2009 Apr 22;10:37. doi: 10.1186/1471-2202-10-37 (PMC2685396; doi:10.1186/1471-2202-10-37)

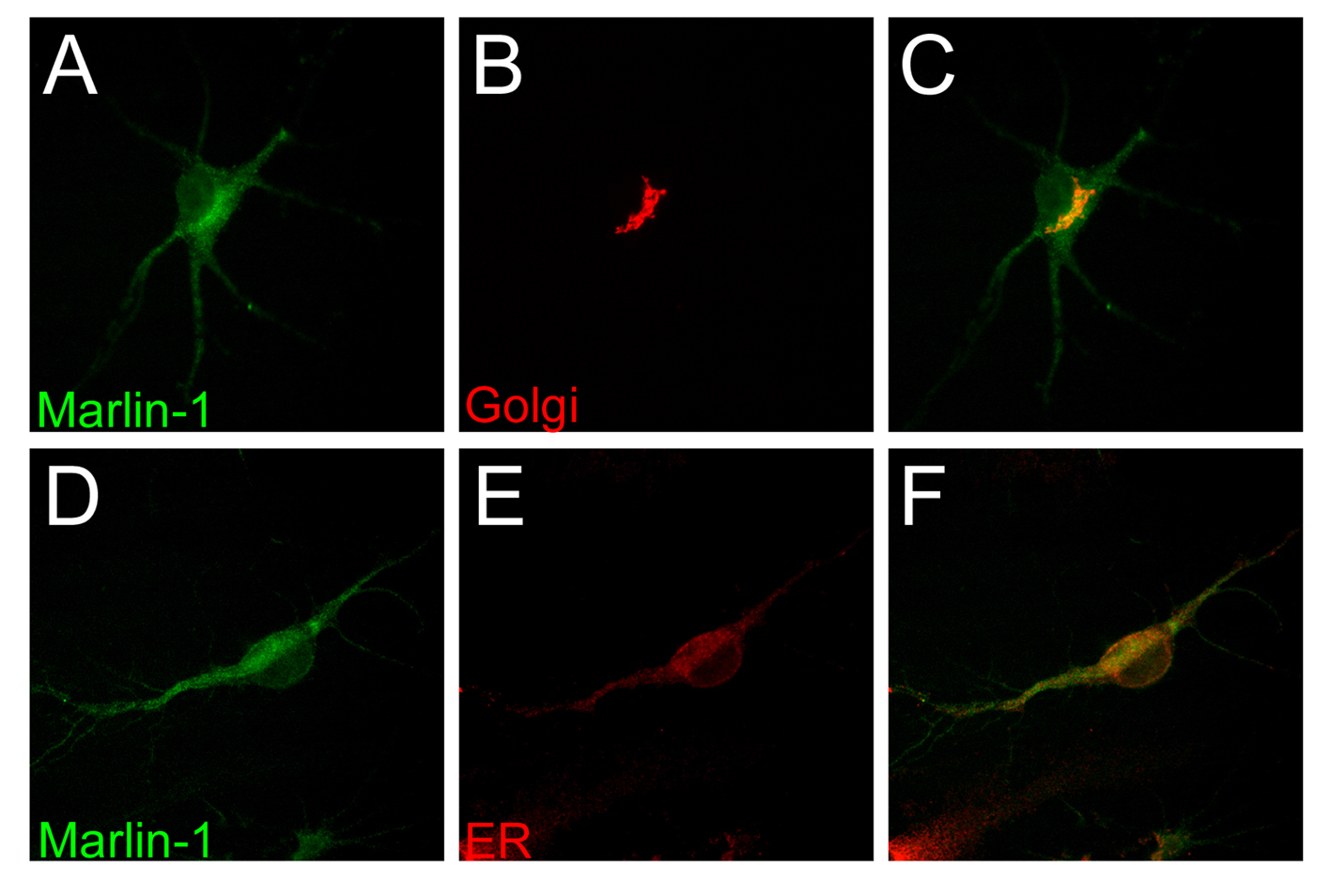

Supplement: Additional file 1 — Subcellular distribution of Marlin-1 in cultured hippocampal neurons relative to secretory organelles. Neurons were fixed at 8 div and stained with Marlin-1 antibodies (A and D) and with Golgi matrix (B) or ER antibodies (E). FITC or TR conjugated secondary antibodies were used to visualize immunostaining. Merge panels are shown on the right (C and F). [file 1471-2202-10-37-S1.tiff]
